# Strategies Adopted by Gay, Bisexual, and Other Men Who Have Sex with Men to Prevent *Monkeypox virus* Transmission — United States, August 2022

**DOI:** 10.15585/mmwr.mm7135e1

**Published:** 2022-09-02

**Authors:** Kevin P. Delaney, Travis Sanchez, Marissa Hannah, O. Winslow Edwards, Thomas Carpino, Christine Agnew-Brune, Kaytlin Renfro, Rachel Kachur, Neal Carnes, Elizabeth A. DiNenno, Amy Lansky, Kathleen Ethier, Patrick Sullivan, Stefan Baral, Alexandra M. Oster

**Affiliations:** ^1^CDC Monkeypox Emergency Response Team; ^2^Rollins School of Public Health, Emory University, Atlanta, Georgia; ^3^Bloomberg School of Public Health, Johns Hopkins University, Baltimore, Maryland.

The first U.S. case of monkeypox during the current outbreak was confirmed on May 17, 2022 (*1*); on August 4, the U.S. Department of Health and Human Services declared the outbreak to be a public health emergency.* To date, most reported monkeypox cases in the United States and globally have occurred among men who reported sexual or close intimate contact with another man during the 3 weeks before symptom onset (*2*). The multipronged response to monkeypox has included expanding access to monkeypox vaccine and developing messaging^†^ for gay, bisexual, and other men who have sex with men (MSM) seeking to reduce their chances for acquiring monkeypox. During August 5–15, 2022, a monkeypox-specific follow-up survey was completed by a convenience sample of 824 MSM who responded to the annual American Men’s Internet Survey (AMIS).^§^ Overall, 48% of respondents reported reducing their number of sex partners, 50% reported reducing one-time sexual encounters, and 50% reported reducing sex with partners met on dating apps or at sex venues since learning about the monkeypox outbreak. Nearly one in five respondents reported receiving ≥1 dose of vaccine to prevent monkeypox. Receipt of vaccine was highest among Hispanic or Latino (Hispanic) men (27.1%) and lowest among non-Hispanic Black or African American (Black) men (11.5%); 17.7% of non-Hispanic White (White) men and 24.2% of men of other race or ethnicity received vaccine. Receipt of vaccine was higher in urban (27.8%) and suburban (14.5%) areas than in other areas (5.7%–7.0%). These data suggest that MSM are taking steps to protect themselves and their partners from monkeypox. It is important that federal, state, and local public health programs continue to deliver tailored, respectful harm reduction messages that do not create stigma to diverse communities of MSM. Vaccine programs should prioritize efforts to maximize equitable access to vaccines to prevent monkeypox.

AMIS is an annual, cross‐sectional, online behavioral survey of a convenience sample of cisgender men in the United States who report sex with another man during the 12 months preceding the survey (*3*). During August 5–15, 2022, AMIS 2021 survey participants who agreed to be recontacted were invited to complete a follow-up survey assessing knowledge of and experiences with monkeypox. After providing research consent, participants answered questions about general knowledge, awareness, and concern about monkeypox; personal behavior changes during the past 3 months because of the monkeypox outbreak; and receipt of vaccine to prevent monkeypox infection. The Emory University Institutional Review Board reviewed and approved procedures for the AMIS survey. This activity was also reviewed by CDC and was conducted consistent with applicable federal law and CDC policy.^¶^

Overall, 2,999 AMIS 2021 participants were invited to participate in the monkeypox survey, and 824 (27.5%) responded and completed all questionnaire sections. Among these respondents, 70.5% were White, and 50.9% were aged <45 years. Most men (90.0%) reported sex with a man during the preceding 3 months (i.e., during the current monkeypox outbreak); 238 (28.9%) reported two or more sex partners during the preceding 14 days. Respondents were from all regions of the United States; (47.8%) lived in urban areas.

Respondents reported changing sexual behaviors since they learned about the monkeypox outbreak (Table 1). Overall, 47.8% reported reducing their number of sex partners, 49.8% reported reducing one-time sexual encounters, and 49.6% reported reducing sex with partners met on dating apps or at sex venues. In addition, 50.4% reported reducing group sex participation, and 41.9% reported reducing attendance at sex venues or social events with close contact because of the monkeypox outbreak.

**TABLE 1:** Strategies for monkeypox prevention adopted by men who have sex with men since they learned about the monkeypox outbreak (N = 797) — American Men's Internet Survey, United States, August 2022

Characteristic (no. who answered not applicable)	No. (%)*		
	Decreased/Less	No change	Increased/More
No. of sex partners (108)	329 (47.8)	358 (52.0)	1 (0.1)
One-time sexual encounters (176)	309 (49.8)	305 (49.2)	6 (1.0)
Sex with a partner met on a dating app or at a sex venue (199)	294 (49.6)	294 (49.6)	5 (0.8)
Having group sex (331)	234 (50.4)	229 (49.4)	1 (0.2)
Going to sex venues or events (407)	162 (41.9)	222 (57.4)	3 (0.8)
Going to social events with close contact, such as dance parties or raves (347)	156 (34.9)	288 (64.4)	3 (0.7)
Use of condoms (275)	6 (1.2)	471 (90.8)	42 (8.1)

* Row percentages calculated after subtracting the number of respondents who reported that the individual behavior was not applicable to them, which is included in parentheses. Row totals including those who felt the item was not applicable might not sum to 797 because of missing data for individual items.

A total of 151 respondents (18.6%) reported receiving ≥1 dose of vaccine to prevent monkeypox (Table 2). Receipt of vaccine was highest among Hispanic men (27.1%) and lowest among Black men (11.5%); 17.7% of White men and 24.2% of men of another race and ethnicity received vaccine. Receipt of vaccine was higher in urban (27.8%) and suburban (14.5%) areas than in medium or small metropolitan (7.0%) or rural (5.7%) areas and was higher in the Northeast (27.8%) and West (21.5%) than in the Midwest (14.9%) or South (13.0%) U.S. Census Regions.

**TABLE 2:** Characteristics of men who have sex with men, by receipt of vaccine to protect against monkeypox — American Men's Internet Survey, United States, August 2022

Characteristic	All participants	Received ≥1 vaccine dose
	No. (column %)	No. (row %)
**Total**	**824 (100.0)**	**151 (18.6)**
**Age group, yrs**		
15–24	46 (5.6)	10 (21.7)
25–34	227 (27.5)	50 (22.6)
35–44	147 (17.8)	35 (24.1)
45–54	128 (15.5)	23 (18.3)
≥55	276 (26.7)	33 (12.0)
Race and ethnicity*		
Black, non-Hispanic	87 (10.6)	10 (11.5)
White, non-Hispanic	581 (70.5)	102 (17.7)
Hispanic or Latino	88 (10.7)	23 (27.1)
Other	68 (8.3)	16 (24.2)
**Health insurance**		
None	24 (2.9)	3 (12.5)
Private only	564 (68.6)	107 (19.2)
Public only	164 (20.0)	25 (15.5)
Other or multiple insurance	70 (8.5)	16 (23.2)
**Population density**		
Urban	394 (47.8)	108 (27.8)
Suburban	188 (22.8)	27 (14.5)
Small/Medium metropolitan	188 (22.8)	13 (7.0)
Rural	54 (6.6)	3 (5.7)
**U.S. Census Bureau region**		
Northeast	174 (21.1)	48 (27.8)
Midwest	141 (17.1)	21 (14.9)
South	305 (37.0)	39 (13.0)
West	204 (24.8)	43 (21.5)
**No. of partners during past 14 days**		
0–1	586 (71.1)	80 (13.9)
2 or more	238 (28.9)	71 (30.1)
**Had group sex with male partners during past 3 months**		
No	580 (70.9)	73 (12.8)
Yes	238 (29.1)	74 (31.5)
**Self-reported HIV status**		
Positive	104 (12.6)	23 (22.3)
Negative	656 (79.6)	123 (19.0)
Unknown	64 (7.8)	5 (7.9)
**Currently taking HIV PrEP^§^**		
No	397 (57.5)	27 (7.0)
Yes	320 (44.6)	98 (31.4)
**Tested for an STI during past 3 months**		
No	410 (50.1)	37 (9.1)
Yes	409 (49.9)	114 (28.2)
**Concerned about getting monkeypox**		
Not concerned or a little concerned	382 (46.9)	47 (12.5)
Somewhat or very concerned	433 (53.1)	104 (24.4)
**Feel confident that they can protect themselves from monkeypox**		
Strongly or mostly disagree	132 (17.7)	13 (10.0)
Strongly or mostly agree	612 (82.3)	136 (22.6)

**Abbreviations:** PrEP = preexposure prophylaxis; STI = sexually transmitted infection.

* The Other category includes non-Hispanic persons of multiple races and Native Hawaiian or other Pacific Islander, American Indian or Alaska Native, and Asian persons.

^†^ Self-reported HIV status was determined from responses to questions about having ever had an HIV test, results of the most recent HIV test, and having ever received a positive HIV test result. Participants’ self-reported HIV status was categorized as positive, negative, or unknown.

^§^ PrEP percentage calculated only among those who reported negative or unknown HIV status (N = 720).

Receipt of vaccine was more common among respondents reporting two or more partners during the preceding 14 days (30.1%) than among those reporting no partners or one partner (13.9%) and among those reporting engaging in group sex with male partners during the preceding 3 months (31.5%) than among those not engaging in group sex during that time (12.8%). Among 662 persons who had not received monkeypox vaccine, 180 (28.5%) indicated that they had tried to get vaccinated.

Frequency of receipt of vaccine was similar for persons who reported having received a diagnosis of HIV infection (22.3%) and those whose most recent HIV test was negative (19.0%). Among those who reported not having HIV, a higher proportion of persons taking HIV preexposure prophylaxis (PrEP) (31.4%) than those not taking HIV PrEP (7.0%) were vaccinated. When limited to 188 men with two or more partners during the preceding 14 days, vaccination was even more prevalent among those taking HIV PrEP: 46 (38.0%) of 121 respondents taking HIV PrEP reported having received vaccine, compared with nine (13.4%) of 67 who were not taking HIV PrEP. Receipt of vaccine was also more prevalent among men who received testing for another sexually transmitted infection during the preceding 3 months.

Three (1.7%) participants reported having received a diagnosis of monkeypox, and 91 (11.4%) of 798 who responded to the question reported knowing someone who had received a diagnosis of monkeypox. Although 53.1% reported they were “somewhat concerned” or “very concerned” about monkeypox, 82.3% reported feeling confident that they could protect themselves from monkeypox.

## Discussion

These findings among a convenience sample of men who reported male sexual contact provide early information about the actions that MSM are taking to reduce their risk for acquiring and transmitting *Monkeypox virus*. These data highlight the importance of health communication in the context of strong community leadership in response to the U.S. monkeypox outbreak. The adoption of prevention strategies reported here aligns with specific harm reduction strategies developed for monkeypox and with broader sexual health information and recommendations for MSM.** A modeling study that assessed the potential effects of reductions in one-time sexual partnerships found that these changes might substantially slow transmission and ultimately reduce the percentage of MSM who acquire monkeypox (*4*). It is important that federal, state, and local public health programs continue to deliver tailored harm reduction messages to diverse communities of MSM. These messages should be designed to reduce the potential for stigma (*5*) and build strength and resiliency (*6*).

These data also suggest racial and ethnic disparities in vaccination, with particularly low reported vaccination among Black men, who are disproportionately affected by monkeypox (*2*). In addition, men who were not taking HIV PrEP or who had not received STI testing were less likely to have received vaccine, suggesting opportunities to improve access for persons who are less engaged with routine health care and sexual health services. Equitable vaccine program implementation involves community engagement in program planning and implementation, engaging diverse partners already working with special populations, delivering vaccines through mobile outreach and pop-up events, and diversifying times and locations for vaccine administration.^††^

These survey data suggest important geographic differences in vaccination, with lower reported vaccination receipt in less urban areas and among men in the South and Midwest. This is particularly concerning because the highest number of cases reported to date have been from southern states.^§§^ Expanding vaccine availability geographically, including diversifying vaccination locations to include nonurban areas, can help ensure that those who need vaccination have access to it. This will be especially important as vaccine availability increases and vaccine strategies expand beyond postexposure prophylaxis to include preexposure vaccination.

The findings in this report are subject to at least four limitations. First, this survey represents a convenience sample of Internet-using cisgender MSM who chose to participate in a survey about monkeypox. This subset of men is older and less racially diverse than the full AMIS sample (*7*), and persons who were more concerned about monkeypox might have been more likely to complete the survey, which could lead to overestimates of behavior modifications and receipt of vaccine. Additional efforts to collect information from populations disproportionately affected by the current monkeypox outbreak are underway. Second, these data are self-reported and might be subject to social desirability bias. Third, the reported number of partners during the preceding two weeks might not reflect sexual behaviors throughout the entire outbreak (and thus eligibility for expanded postexposure prophylaxis with vaccine), particularly if behaviors changed because of the outbreak or receiving vaccine; ongoing monitoring will be needed to understand persistence or changes in these findings over time. Finally, because the survey did not ask whether respondents had seen harm reduction messaging, these changes cannot be ascribed directly to messaging efforts.

Addressing inequities in vaccine availability and coverage is an urgent public health priority. However, vaccination alone will not be sufficient to end the current monkeypox outbreak. These findings suggest that MSM are already taking actions to protect their sexual health and making decisions to reduce risk to themselves and their partners. These changes are important to protect MSM from exposure before access to vaccine is possible and after vaccination.^¶¶^ CDC will continue to work with state and local partners to develop and provide tailored, respectful harm reduction messaging to diverse communities affected by the monkeypox outbreak and to monitor the impact of messaging and prevention strategies, including vaccination.

**Summary**

**What is already known about this topic?**

A global monkeypox outbreak is currently primarily affecting gay, bisexual, and other men who have sex with men.

**What is added by this report?**

In a recent survey of gay, bisexual, and other men who have sex with men, approximately one half reported reducing their number of sex partners, one-time sexual encounters, and use of dating apps because of the monkeypox outbreak. Receipt of vaccine to protect against monkeypox varied by race, ethnicity, and geography.

**What are the implications for public health practice?**

It is essential that public health programs continue to deliver tailored, respectful harm reduction messages that do not create stigma to diverse communities of men who have sex with men. Vaccine programs should prioritize efforts to maximize equitable access.

## Footnotes

* https://www.hhs.gov/about/news/2022/08/04/biden-harris-administration-bolsters-monkeypox-response-hhs-secretary-becerra-declares-public-health-emergency.html (Accessed August 25, 2022).

† This messaging was first published online June 7, 2022, and was updated as of August 5, 2022. https://www.cdc.gov/poxvirus/monkeypox/sexualhealth/index.html (Accessed August 25, 2022).

§ https://emoryamis.org (Accessed August 25, 2022).

¶ 45 C.F.R. part 46.102(l)(2); 21 C.F.R. part 56; 42 U.S.C. Sect. 241(d); 5 U.S.C. Sect. 552a; 44 U.S.C. Sect. 3501 et seq.

** https://www.cdc.gov/msmhealth/for-your-health.htm (Accessed August 25, 2022).

†† https://www.cdc.gov/poxvirus/monkeypox/interim-considerations/overview.html#equity (Accessed August 25, 2022).

§§ https://www.cdc.gov/poxvirus/monkeypox/response/2022/us-map.html (Accessed August 25, 2022).

¶¶ The current vaccine regimen for JYNNEOS vaccine consists of 2 doses, 28 days apart, with maximal immune protection achieved 2 weeks after the second dose: https://www.cdc.gov/poxvirus/monkeypox/vaccines.html. Persons who are vaccinated should continue to take steps to protect themselves from monkeypox as knowledge of vaccine efficacy during the current outbreak continues to evolve: https://www.cdc.gov/poxvirus/monkeypox/considerations-for-monkeypox-vaccination.html (Accessed August 25, 2022).
